# Research trends in Korean medicine based on temporal and network analysis

**DOI:** 10.1186/s12906-019-2562-0

**Published:** 2019-07-05

**Authors:** Sang-Kyun Kim, Yongtaek Oh, SeJin Nam

**Affiliations:** 1 Future Medicine Division, Korea Institute of Oriental Medicine, 1672 Yuseong-daero, Yuseong-gu, Daejeon, 34054 Republic of Korea; 2 Department of Diagnostics, College of Korean Medicine, Woosuk University, Jeonju, 54986 Republic of Korea; 3 LifeSemantics, 636, Gangnam-daero, Gangnam-gu, Seoul, 06034 Republic of Korea

**Keywords:** Korean medicine, OASIS, Temporal analysis, Network analysis, Trend analysis

## Abstract

**Background:**

Much research on Korean medicine has been recently published in Korea. The aim of this study was to determine the research trends in Korean medicine by performing a comprehensive analysis of articles that have been published in Korea using temporal and network analysis methods.

**Methods:**

A total of 29,876 articles from 1963 to 2018 were prepared from OASIS (Oriental Medicine Advanced Searching Integrated System), the largest portal for Korean medicine. After the keywords and years were extracted from the metadata of the articles, an annual frequency matrix was obtained for the keywords. By using the matrix, the temporal trends of the keywords were analyzed by comparing the changes in similarity between the lists of keywords by year. Moreover, to analyze the relationship among research topics, a clustered network was constructed in which a node was a keyword and an edge was a similarity between two keywords.

**Results:**

The temporal trend of the keywords was classified into six chronological phases. The appearance frequency of most keywords tended to increase gradually, but only the keywords “mibyeong,” “systems biology” and “korean medicine hospital” appeared in the most recent phase. The network of keywords was clustered and visualized into thirteen groups with the Gephi software. The main keywords in each group were related to effects such as “anti-inflammation” and “antioxidant,” to diseases such as “allergic rhinitis” and “diabetes” and to therapies such as “herbal acupuncture” and “herbal formula.”

**Conclusions:**

The analysis of the trends determined in this study provides a systematic understanding as well as future research directions in Korean medicine to researchers. In the future, an overall analysis of the research trends in Korean medicine will be done by analyzing articles published in Korea and other countries.

**Electronic supplementary material:**

The online version of this article (10.1186/s12906-019-2562-0) contains supplementary material, which is available to authorized users.

## Background

OASIS (Oriental Medicine Advanced Searching Integrated System, https://oasis.kiom.re.kr) is a portal that provides research information on Korean medicine, which refers to the traditional medicine practices that originated and developed in Korea [1] (https://en.wikipedia.org/wiki/Traditional_Korean_medicine). The information includes about 29,000 articles on Korean medicine published by 55 academic journals since 1963 in Korea, which is the largest literature database in Korea for Korean medicine. Most systematic reviews and meta-analyses on herbal medicines and therapies in Korean medicine have included searches in the OASIS as well as PubMed, EMBASE, and CNKI [2–4]. Recently, the number of articles on Korean medicine published in Korea has been increasing. Because OASIS has been accumulating these articles for a long time, it is worth analyzing them. A study was recently done to compare the search trends of OASIS between experts and the general public but that study did not analyze the research trends in the published articles found in OASIS [5]. This study analyzed the research trends in Korean medicine using the articles found in OASIS. Statistically analyzing research trends in published studies can help researchers identify current research interests as well as provide future research directions.

Generally, to analyze research papers, categories are first created that classify research topics after having extracted them manually from articles or through text mining [6–8]. Then, a researcher quantitatively analyzes when a specific research topic was studied as well as to what degree and assigns it to a category. Recently, studies have proposed analyzing research trends using SNA (Social Network Analysis) [9, 10], which is a method that investigates social structures based on networks and graph theory [11]. In this approach, clustering is performed on the social network, in which a node is a research topic, and an edge is a relationship between two research topics. They present insights into research trends by analyzing the relationship among research topics.

The aim of this study was to analyze the overall research trends in Korean medicine based on temporal and network analysis methods. To this end, after extracting keywords and publication years from Korean medicine articles, we first analyzed the change in the appearance frequency of keywords over time. Moreover, a clustered network was constructed with the keywords, and the research trends were determined by analyzing each group in the cluster. Based on the analyses of these keywords, this study provides comprehensive insights as well research directions in Korean medicine.

## Methods

### Data preparation

**Fig. 1:**
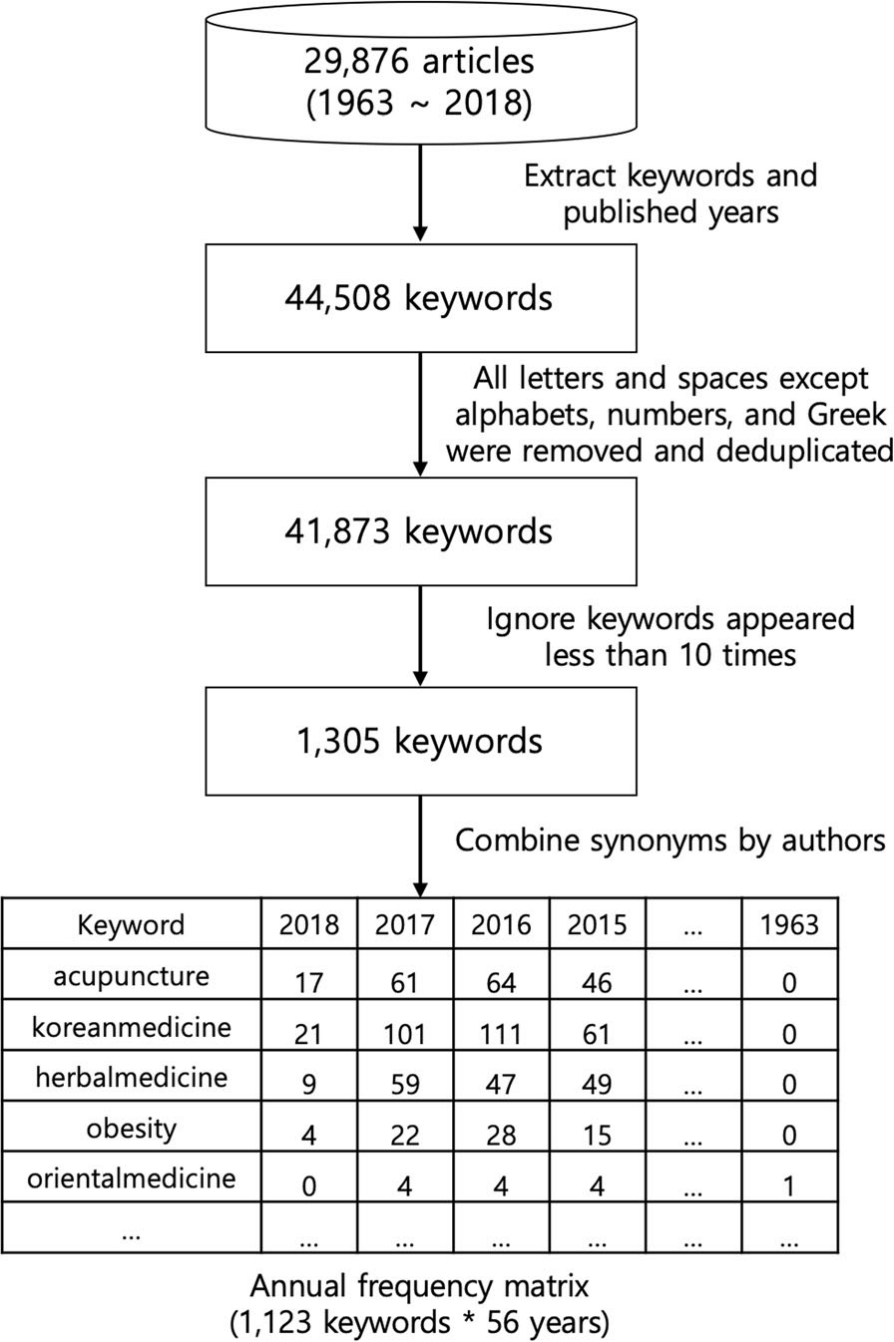
Process for constructing the annual frequency matrix of the keywords. A matrix consisting of 1123 keywords over 56 years was constructed from 29,876 articles

OASIS currently provides the metadata and full-text of 29,876 articles which have been published from 1963 to 2018 in Korea. To analyze the research trends in Korean medicine, the metadata of these articles were extracted directly from the OASIS website. Because articles have unique numbers within a certain range in OASIS, we have downloaded their metadata individually using the HTTP GET method. After the keywords and publication years were extracted from the metadata of the articles, a two-dimensional matrix was obtained for the appearance frequency of 44,508 keywords from 1963 to 2018, and frequency refers to the number of articles containing each keyword. To combine the arrays of the synonyms of keywords into one, the following steps were carried out. First, all letters and spaces except for alphabetic letters, numbers, and Greek letters were removed, and all alphabetic characters were made into lowercase. After combining the duplicates of keywords, the number of different keywords was 41,873, and the number of keywords that appeared more than 10 times was 1305. We ignored the keywords that appeared less than 10 times, and two of the study’s authors reviewed the 1305 keywords to combine the synonyms and reached a consensus. Cohen’s kappa for inter-rater agreement between the two authors was 0.87, which is a substantial agreement. Finally, we obtained an annual frequency matrix for 1123 keywords. Figure 1 shows the process for constructing the matrix. The full list of about 41,873 keywords used in our study is provided in Additional file 1. All spaces, hyphens, and apostrophes between words were removed in the data analysis, but we added them back in this article for readability.

### Data analysis

The temporal trend of the research topics was analyzed based on the frequency of the keywords by year. The basic idea is that if a keyword is used frequently for a given period, then the keyword is the main research topic during that timeframe. There are many ways to calculate the correlation between lists of keywords per year. In this study, the similarity between two frequencies of keyword lists by year was calculated with the weighted Jaccard similarity [12]. The similarity between **S** and **T** is expressed by the following equation, given two frequency lists **S** and **T**. *S*_*k*_ and *T*_*k*_ represent the *k*th value of **S** and **T**, respectively. This equation is also used in the network analysis and temporal analysis.

$$ J\left(\mathbf{S},\mathbf{T}\right)=\frac{\sum_k\min \left({S}_k,{T}_k\right)}{\sum_k\max \left({S}_k,{T}_k\right)} $$

In the temporal analysis, the Jaccard similarity is calculated between the column data lists of the matrix in Fig. 1. For example, when calculating the similarity value between the two frequency lists of “2018” and “2017”, the input values are S = {17, 21, 9, 4, 0, ...} and T = {61, 101, 59, 22, 4, ...}; thus, the similarity value is *J*(**S**, **T**) = (17 + 21 + 9 + 4 + 0 + …)/(61 + 101 + 59 + 22 + 4 + …) shown in Fig. 1. In this paper, we investigated the change in the weighted Jaccard similarities between the frequencies of the keyword lists from 1963 to 2018 and analyzed which keywords mainly brought about this change. The similarity value is 1.0 for perfect agreement between two frequency lists and 0 for disjointed frequency lists. If keywords appear with a similar frequency in 1 year and the previous year, a high similarity value is returned.

In addition to the analysis of the frequency of keyword lists by year, the correlation between annual frequency lists of keywords by keywords was also analyzed. First, a network of keywords was constructed, in which a node is a keyword in the articles, and an edge is the value of the weighted Jaccard similarity between the annual frequency lists of two keywords. When two keywords were used together more frequently in the same year, the obtained similarity value increased. In the network analysis, the Jaccard similarity is calculated between the row data lists of the matrix seen in Fig. 1. For example, when calculating the similarity value between “acupuncture” and “korean medicine”, the input values are S = {17, 61, 64, 46, ..., 0} and T = {21, 101, 111, 61, ..., 0}; thus, the similarity value is *J*(**S**, **T**) = (17 + 61 + 64 + 46 + … + 0) / (21 + 101 + 111 + 61 + … + 0) shown in Fig. 1. This network of keywords was explored and visualized with Gephi 0.8.2 [13], an open-source software for graphs and networks. To reveal the association among the nodes in the network, node clustering is necessary. In this paper, we used the MCL (Markov Cluster) algorithm [14] which is able to cluster the weighted network. Because the algorithm is implemented as a plugin of Gephi (https://gephi.org/plugins/#/plugin/markov-cluster-algorithm-mcl), the association between the nodes in the network can be easily analyzed.

## Results

### Frequency of the keywords

Before the analysis of the research trends shown by the keywords, we identified the most frequently used keywords. Table 1 shows the top 30 most frequently used keywords among the 1123 keywords. The most frequently used keyword was “acupuncture” followed by “korean medicine,” “sasang constitution,” “herbal medicine,” and “obesity.” The full list with the frequency for the 1123 keywords is provided in the Additional file 1.

**Table 1:** List of the frequencies of the keywords in OASIS. Abbreviations: cox-2 (cyclooxygenase-2); no (nitric oxide); tnf-α (tumor necrosis factor α)

No	Keyword	Frequency
1	acupuncture	1153
2	korean medicine	870
3	sasang constitution	671
4	herbal medicine	648
5	obesity	497
6	oriental medicine	440
7	stroke	378
8	anti-inflammation	377
9	apoptosis	374
10	no	369
11	antioxidative activity	325
12	cytokine	318
13	atopic dermatitis	299
14	electroacupuncture	293
15	visual analogue scale	289
16	moxibustion	284
17	heart rate variability	271
18	dongeuibogam	253
19	herbal acupuncture	246
20	antioxidant	235
21	tnf-α	223
22	low back pain	211
23	pharmacopuncture	209
24	bell’s palsy	202
25	hyperlipidemia	194
26	oriental medical therapy	175
27	diabetes	173
28	toxicity	171
29	cox-2	167
30	inflammation	166

### Trend analysis by year

**Fig. 2:**
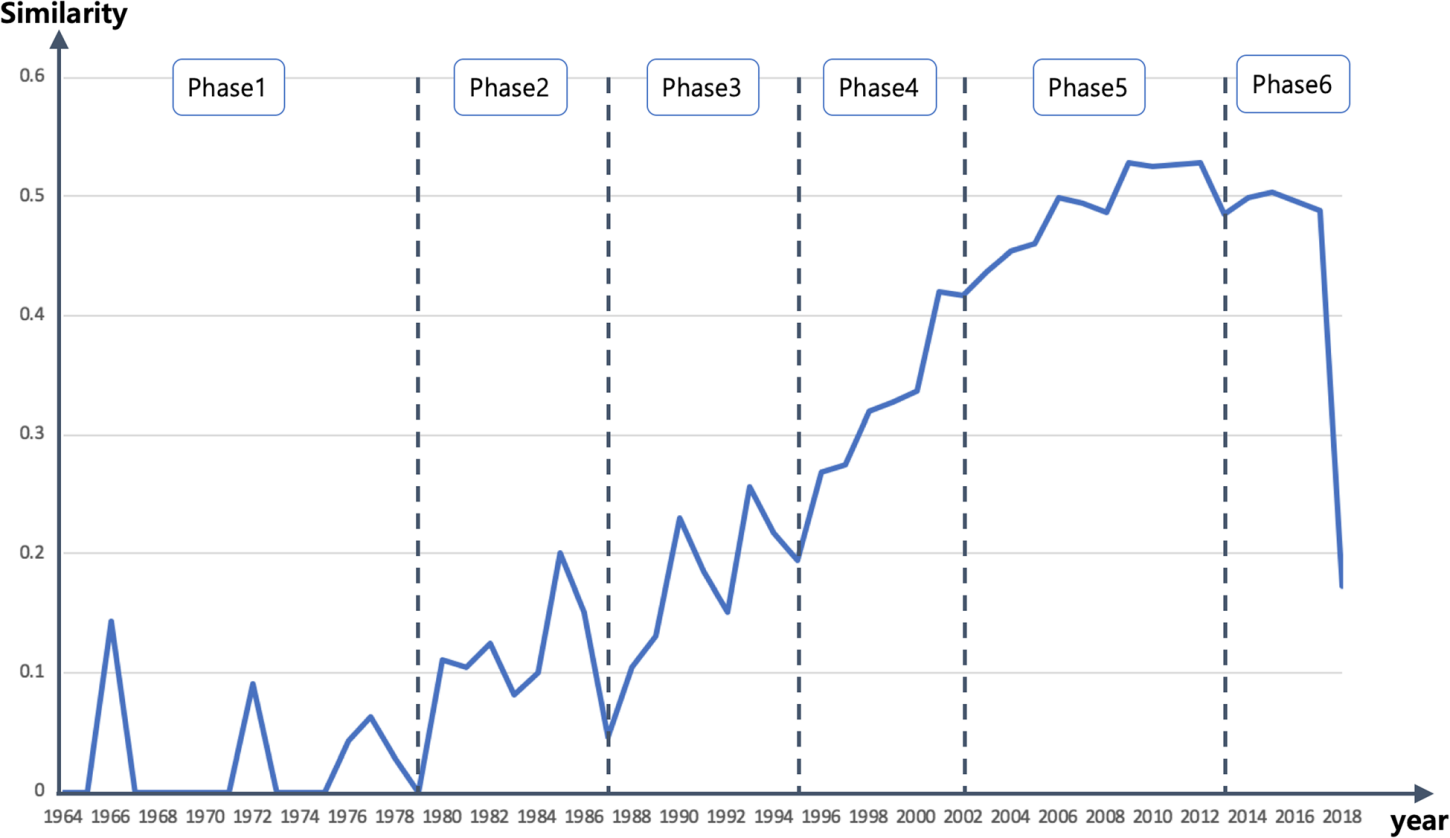
Chart of changes in the similarity of the keywords by year. The changes in the weighted Jaccard similarities between two frequency lists of keywords in a year and the previous one are shown

Temporal research trends were analyzed based on the change in the appearance frequency of the keywords by each year. To examine the temporal change of the keywords, we calculated the similarity of the lists of keywords by year. Figure 2 shows the changes in the weighted Jaccard similarities between two frequency lists of keywords in a year and the previous one.

To analyze the temporal research trends, the years were divided into six chronological phases. Phase 1 consisted of 1963 ~ 1978 because within this phase, there were many years with a similarity of zero. The cut-off point, at which the similarity starts to increase after a sharp decrease, indicates a significant change in a research trend. Therefore, the years 1979 to 2018 were divided based on the values of the similarity. There were, however, two sharp decreasing intervals between 1987 and 1995, but each interval was too short to be divided into its own phase. From 1995 to 2013, there was no sharp decrease, but there were long intervals; hence, we divide these years into phase 4 and phase 5.

Table 2 lists the appearance frequencies of the keywords which show a different change distribution compared to the other keywords between each phase. The total number of articles in each phase is provided at the top of Table 2. The full list of the appearance frequencies for the keywords is given in the Additional file 1.

**Table 2:** Examples of the appearance frequencies of the keywords in each phase

Keywords	Phase1 (1963~1978)	Phase2 (1979~1986)	Phase3 (1987~1994)	Phase4 (1995~2001)	Phase5 (2002~2012)	Phase6 (2013~2018)
	643	498	1889	5758	15,921	5167
acupuncture	6	10	29	140	653	315
oriental medicine	8	7	17	74	299	35
korean medicine	0	0	0	4	314	552
aqua acupuncture	4	2	10	48	9	9
pharmacopuncture	0	0	0	0	89	120
cancer	1	2	1	12	58	22
lung cancer	0	0	1	6	20	22
breast cancer	0	0	1	3	26	15
gastric cancer	0	0	0	2	6	8
colorectal cancer	0	0	0	3	4	5
sasang constitution	5	0	17	63	413	173
soyangin	0	0	2	11	64	47
taeeumin	0	0	6	8	50	35
stress	3	0	5	19	74	18
anti-inflammation	0	12	10	17	214	124
pattern identification	0	0	2	2	77	80
psoriasis	0	0	1	3	24	26
alopecia	0	0	2	1	18	20
korean medical treatment	0	0	0	3	9	107
traditional chinese medicine	0	0	0	6	61	65
review	0	0	0	2	39	76
systematic review	0	0	0	0	47	73
case report	0	0	0	0	15	54
clinical practice guideline	0	0	0	0	5	42
integrative medicine	0	0	0	0	8	27
meta-analysis	0	0	0	0	7	27
toxicity test	0	0	0	0	8	21
mibyeong	0	0	0	0	0	14
systems biology	0	0	0	0	0	11
korean medicine hospital	0	0	0	0	0	15
endotoxin	1	0	16	8	8	0
intravascular coagulation	0	2	15	7	0	0
antioxidant	0	0	1	14	155	65
lipid peroxidation	0	0	0	45	37	8
lipid peroxide	0	0	1	20	6	0
xanthine oxidase	0	0	0	25	13	2
superoxide dismutase	0	0	0	13	9	3

In Table 1, the appearance frequency of “acupuncture,” which has the highest frequency, tends to increase continuously until phase 5 and then decrease in Phase 6, which is similar to the changes in the chart shown in Fig. 2. The appearance frequency of most of the keywords changes in a similar manner as the pattern for “acupuncture.” In addition to this general change pattern, there are the following unusual change patterns.

Any two keywords with similar meanings were changed in their frequencies over time. For example, the keyword “oriental medicine” increased until Phase 5 and then rapidly decreased in Phase 6 while “korean medicine” increased rapidly from Phase 5. In 2012, the official name of Korean medicine changed from “oriental medicine” to “korean medicine” [15]. Therefore, its frequency in articles seems to have changed. The keyword “aqua acupuncture” was mostly used up until phase 4, and then, “pharmacopuncture” emerged as the new keyword since Phase 5. This shows that research on “aqua acupuncture” changed to research on “pharmacopuncture” [16].

Some keywords belong to one research topic, appearing at different times. For example, research on “cancer” in Korean medicine has been carried out continuously, but “lung cancer” and “breast cancer” only appeared since Phase 3 and “gastric cancer” and “colorectal cancer” since Phase 4. Research on “sasang constitution” has been done since Phase 1, but “soyangin” and “taeeumin,” only appeared since Phase 3. Sasang constitution is a traditional typological constitution in Korean medicine. It classifies people into four types, “soyangin,” “soeumin,” “taeyangin” and “taeeumin,” based on the eum-yang (yin-yang in Chinese) theory. Two types of sasang constitution were mentioned in the text of the articles, but they did not appear as keywords until Phase 3.

Most of the keywords had the highest appearance frequency in Phase 5, but the following keywords were used more in Phase 6 than in Phase 5. “pattern identification,” “psoriasis,” and “alopecia” have appeared since Phase 3; “korean medical treatment,” “traditional chinese medicine,” and “review” have appeared since Phase 4, and “systematic review,” “case report,” “clinical practice guideline,” “integrative medicine,” “meta-analysis,” and “toxicity test” have appeared since Phase 5. In particular, “mibyeong,” “systems biology,” and “korean medicine hospital” did not appear until Phase 5, and only began to appear from Phase 6.

Contrary to the increasing frequency in recent years, there were keywords that appeared quite often in the past and then decreased in recent years. “endotoxin” and “intravascular coagulation” and “lipid peroxidation,” “lipid peroxide,” “xanthine oxidase,” and “superoxide dismutase” occurred many times in Phase 3 and Phase 4, respectively, and then, their appearances decreased. Moreover, there were also keywords that showed an increase or decrease only in a specific phase. “sasang constitution” and “stress” were not used in phase 2, and “anti-inflammation” started to be used abruptly since Phase 2.

### Network clustering of research topics

**Fig. 3:**
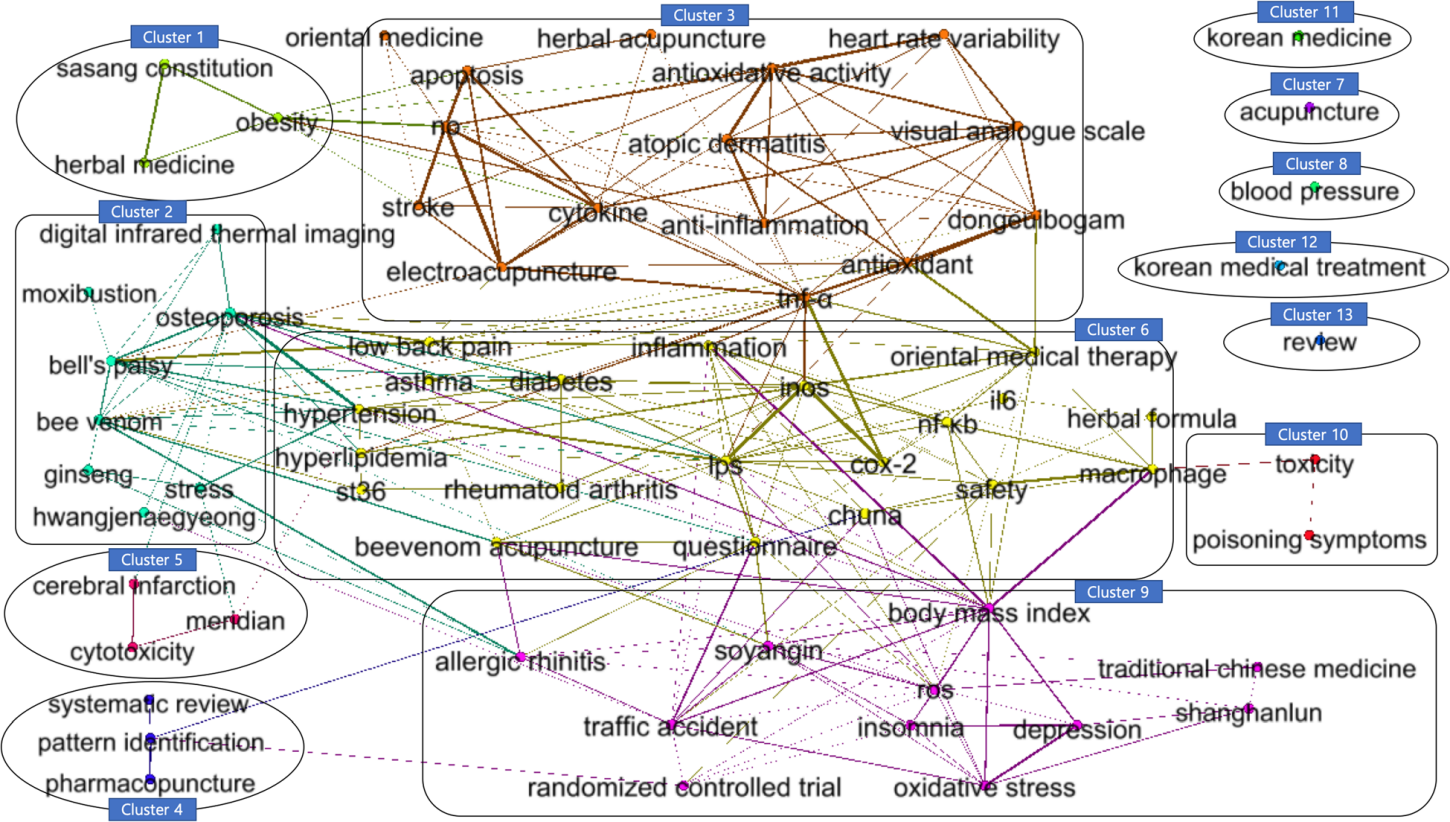
Thirteen groups of research topics. The network consists of 70 nodes and 245 edges, which are clustered into nine groups using the MCL algorithm. Abbreviations: il6 (interleukin 6); inos (inducible nitric oxide synthase); lps (lipopolysaccharide); nf-κb (nuclear factor kappa-light-chain-enhancer of activated B cells); ros (reactive oxygen species)

Figure 3 shows a clustered network of the keywords visualized using Gephi. The network consists of 70 nodes and 245 edges containing only nodes with a frequency of 100 or more and only edges with a weighted Jaccard similarity of 0.57 or more. They are clustered into thirteen groups using the MCL algorithm with parameter values of power = 2.0, inflation = 2.0 and pruning = 0.02. The edge between two nodes is the similarity of the frequencies of two keywords by year. The higher the similarity, the thicker the thickness of the edge.

Table 3 lists the nodes that belong to each cluster. Most of the nodes are clustered in cluster 2, 3, 6, and 9, and there are connections among the clusters. However, five clusters of 7, 8, 11, 12, and 13 exist alone without any connections to the other clusters. All cluster images except for the clusters with one node are shown in the Additional file 1: Figures S1 to S8.

**Table 3:** Distribution of the nodes in each cluster

Clusters	Nodes
Cluster 1	“herbal medicine,” “obesity,” and “sasang constitution”
Cluster 2	“bee venom,” “bell’s palsy,” “digital infrared thermal imaging,” “ginseng,” “hwangjenaegyeong,” “moxibustion,” “osteoporosis,” “stress”
Cluster 3	“anti-inflammation,” “antioxidant,” “antioxidative activity,” “apoptosis,” “atopic dermatitis,” “cytokine,” “dongeuibogam,” “electroacupuncture,” “heart rate variability,” “herbal acupuncture,” “no,” “oriental medicine,” “stroke,” “tnf-α,” “visual analogue scale”
Cluster 4	“pattern identification,” “pharmacopuncture,” “systematic review”
Cluster 5	“cerebral infarction,” “cytotoxicity,” “meridian”
Cluster 6	“asthma,” “bee venom acupuncture,” “chuna,” “cox-2,” “diabetes,” “herbal formula,” “hyperlipidemia,” “hypertension,” “il6,” “inflammation,” “inos,” “low back pain,” “lps,” “macrophage,” “nf-κb,” “oriental medical therapy,” “questionnaire,” “rheumatoid arthritis,” “safety,” “st36”
Cluster 7	“acupuncture”
Cluster 8	“blood pressure”
Cluster 9	“allergic rhinitis,” “body mass index,” “depression,” “insomnia,” “oxidative stress,” “randomized controlled trial,” “ros,” “shanghanlun,” “soyangin,” “traditional chinese medicine,” “traffic accident”
Cluster 10	“poisoning symptoms,” “toxicity”
Cluster 11	“korean medicine”
Cluster 12	“korean medical treatment”
Cluster 13	“review”

## Discussion

This study analyzed the research trends in Korean medicine using temporal and network analysis. To investigate the temporal research trends, we examined how the similarities between the frequencies of the keywords by year change and how frequent the keywords appeared in the six chronological phases. Most of the keywords appeared most frequently in Phase 5, not Phase 6. This is because the period for Phase 5 is longer than that for Phase 6. Nevertheless, the appearance frequency of some of the keywords was the highest in Phase 6. Thus, these keywords reflect the recent research topics in Korean medicine. Moreover, it can be expected that many studies will also be conducted in relation to the keywords in the future.

In particular, “mibyeong,” “systems biology,” and “korean medicine hospital” began to emerge in Phase 6. Mibyeong is a concept meaning the sub-health state [17]. Recently, because preventive medicine has been emphasized in Korea, research projects on mibyeong have emerged, and papers have been published through these research projects [18–20]. Systems biology has been studied extensively in various fields since 2000. In the field of Korean medicine, systems biology has recently been investigated. Because Korean medicine promotes integrative medicine [21], research on systems biology is expected to increase in the future. In Korea, EMR (Electronic Medical Record) systems have recently been implemented at Korean medicine hospitals, and significant amounts of patient data are now stored in these systems [22]. Clinical studies on patients in Korean medicine hospitals are being conducted, including retrospective ones, using EMR data [23]. Because high-quality clinical data have been accumulated over time, clinical studies on Korean medicine will increase.

There was much research in the past on the following keywords, but it has decreased recently.

The keywords “endotoxin,” “intravascular coagulation,” “lipid peroxidation,” “lipid peroxide,” “xanthine oxidase,” and “superoxide dismutase” appeared frequently in Phase 3 or Phase 4 and decreased since then. This is due to the concentration of specific research methods over a specific period of time. In Phase 3, there were many studies on the effects of herbal formulas on intravascular coagulation induced by endotoxin in rats [24, 25]. There were many of these studies done by researchers. As shown by the increase in the keyword “antioxidant” in Table 2, studies on antioxidant effects have been done continuously. However, after reviewing the articles with the keywords “lipid peroxidation,” “lipid peroxide,” “xanthine oxidase,” and “superoxide dismutase,” it was observed that several researchers used research methods that were related to these keywords in Phase 4 [26, 27].

In this study, to analyze the temporal trend of the articles, all the years were divided into six chronological phases. However, this division is an ad hoc method, and there are other various analytical methods. For example, the years can be divided into five- or ten-year periods. The appearance frequencies of the keywords by five- and ten-year periods are provided as Additional file 1. When the keywords were divided by 5-year periods, the change in trend was small, and clear trends were not observed. When they were divided into 10-year periods, the change in trend was similar to the results of Fig. 2, but the interval in Phase 6 became longer, and the frequencies of the keywords in phase 6 increased. In Table 2, the frequency of “oriental medicine” was “299” and “35” in Phase 5 (2002 ~ 2012) and Phase 6 (2013 ~ 2018), respectively. In the table divided by 10-year periods, there was no change in the frequency for “oriental medicine” between Phase 5 (1999 ~ 2008) and Phase 6 (2009 ~ 2018) each with a value of “185.” This is because the Korean Oriental Medicine Association announced that the official name for oriental medicine changed from “oriental medicine” to “korean medicine” in 2012.

To analyze the research trends by the network analysis, thirteen groups were clustered. In fact, the edge between two nodes in the network does not indicate a direct relationship between two keywords in the articles. However, if the appearance frequency of two keywords are similar in years, it can be considered that they have a similar research trend.

Cluster 1 contains three keywords: “herbal medicine,” “sasang constitution,” and “obesity.” It is well known that taeeum-type people in sasang constitutional medicine are associated with “obesity” [28, 29]. Cluster 1 is the group with these studies.

The main research topics in cluster 2 include “moxibustion,” “bell’s palsy,” “digital infrared thermal imaging,” “ginseng,” and “stress.” In Korean medicine, the effects of moxibustion in treatment of bell’s palsy have been studied [30], and digital infrared thermal imaging has been used to evaluate the effects before and after the treatment of bell’s palsy [31]. It is well known that ginseng has marked effects including stress [32]. Moxibustion has traditionally been used as an important treatment in Korean medicine with acupuncture; however, in cluster 2, the relationships between moxibustion and the other nodes are weak. This indicates that there is a lack of research on the effect of moxibustion. Future studies are needed on the effect of moxibustion in various diseases as well as in bell’s palsy.

Cluster 3 contains the research topics “anti-inflammation,” “antioxidant,” and “apoptosis” for herbal medicines. There is no direct connection with medicinal materials and formulas, but it was observed that studies on their anti-inflammatory, antioxidant, and anti-apoptotic effects are clustered. Moreover, research on the effects of “electroacupuncture” and “herbal acupuncture” has been on therapy for diseases such as “stroke.”

The main research topics in cluster 6 include “diabetes,” “hypertension,” “hyperlipidemia,” “herbal formula,” “lps,” “inos,” “cox-2,” “macrophage,” “bee venom acupuncture” and “safety.” “herbal formula” was found in research on treating metabolic diseases such as “diabetes,” “hypertension,” and “hyperlipidemia” and on evaluating its safety. In addition, it is related to research methods for inflammation that included “lps,” “inos,” “cox-2,” and “macrophage.” Recently, “safety” has become increasingly important for bee venom or herbal medicines [33]; however, there is no relationship between “safety” and “bee venom acupuncture” while that between “safety” and “herbal formula” is weak. Further studies are need to prove the safety of herbal medicines or pharmacopuncture in Korean medicine.

Cluster 3 and cluster 6 have similar characteristics in that they have research topics on the effect of herbal medicines. The two clusters are linked through keywords such as “antioxidant” and “tnf-α,” but they are divided into different clusters due to different disease groups.

The main research topics of cluster 9 are “oxidative stress,” “ros,” “allergic rhinitis,” “depression,” and “insomnia.” “oxidative stress” refers to cell damage due to increased intracellular levels of “ros” and is known as one of the major causes of “allergic rhinitis” [34]. Oxidative stress is also related to psychological disorders such as “depression” and “insomnia” [35, 36]. “randomized controlled trial” is an important method in clinical research [37], and many studies have used this method for Korean medicine. However, it was shown that the relationship between “randomized controlled trial” and other research topics is weak. Therefore, “randomized controlled trial” for the many diseases treated by Korean Medicine need to be adopted in the future.

In clusters 7 and 11, the frequency of “acupuncture” and “korean medicine” was much higher compared to other keywords; thus, that it could not be clustered with the other research topics. “blood pressure” in cluster 8, “korean medical treatment” in cluster 12, and “review” in cluster 13 also have a low similarity with the other research topics. Clusters 1, 4, and 5 consist of three nodes, which have weak connections with the nodes in the other clusters as well as within the cluster. Cluster 10 contains research topics on symptoms due to toxic substances.

In this study, we analyzed the research trend of Korean medicine in Korea using the OASIS database, which provides domestic articles in the Korean medicine field. However, research on Korean medicine is being published in both domestic and overseas journals. At present, it is not easy to search for only Korean medicine studies published overseas. If research trends can be investigated with overseas journals, a more systematic analysis of the research trends will be possible.

Due to these difficulties, trends in similar research fields such as TCM were commonly analyzed with a limited scope. Wang et al. analyzed the articles published in the American Journal of Chinese Medicine between 2004 and 2011 [38]. They explained that herbal medicine studies accounted for more than 60% of the total published studies, and cancer studies showed an increasing tend during this period. Chen et al. analyzed the research trends of traditional Chinese medicine formulas after searching for formulas published between 2000 and 2016 in the Web of Science database [39]. Formula studies published during this period accounted for 63% of the total articles, and the treatment of diseases such as cardiovascular diseases and cancers were mostly discussed. In the present study, the keyword “herbal medicine” was the fourth most frequently used keyword shown in Table 1. Moreover, this study, unlike the two studies mentioned above, did not categorize the disease types; therefore, it cannot be directly compared. However, it was found that research on various diseases including cancer is increasing. These results suggest that traditional medical studies including TCM and Korean medicine have some similar research trends. In the future, it is necessary to conduct deeper studies using various methods in the field of traditional medicine.

## Conclusions

This study presented the research trends in Korean medicine by analyzing articles available in OASIS. To extract the research trends, after constructing a matrix for the appearance frequencies of keywords by year, the temporal changes and a clustered network of keywords were analyzed using the matrix. We identified the main research topics by year and proposed future studies based on the temporal changes in the appearance frequencies of the keywords. In addition, by analyzing the relationship of the keywords based on the similarity between them, we determined future research directions necessary for Korean medicine. In the future, we will investigate the overall research trends of Korean medicine by analyzing articles published in Korea as well as those published overseas.

## Additional file

Additional file 1: **Keyword**. Full list of 41,873 keywords used in our study. **Table 1**. Full list of 1,123 keywords and their frequencies. **Table 2**. Full list of the appearance frequencies for the keywords. **Figure S1-S8**. All cluster images. **Table2-5y**. Appearance frequencies of the keywords by five-year periods. **Table2-10y**. Appearance frequencies of the keywords by ten-year periods. (ZIP 8463 kb)

## Data Availability

The datasets used and analyzed in the current study are available from the corresponding author upon reasonable request. All data generated or analyzed during this study are included in this published article.

## Abbreviations

cox-2: cyclooxygenase-2
EMR: Electronic Medical Record
il6: interleukin 6
inos: inducible nitric oxide synthase
lps: lipopolysaccharide
MCL: Markov Cluster
nf-κb: nuclear factor kappa-light-chain-enhancer of activated B cells
no: nitric oxide
OASIS: Oriental Medicine Advanced Searching Integrated System
ros: reactive oxygen species
SNA: Social Network Analysis
tnf-α: tumor necrosis factor α
