# Supplementary material for: Research trends in Korean medicine based on temporal and network analysis
Source: BMC Complement Altern Med. 2019 Jul 5;19:160. doi: 10.1186/s12906-019-2562-0 (PMC6612192; doi:10.1186/s12906-019-2562-0)
Supplement: Supplementary file 1 — Keyword. Full list of 41,873 keywords used in our study. Table 1. Full list of 1,123 keywords and their frequencies. Table 2. Full list of the appearance frequencies for the keywords. Figure S1-S8. All cluster images. Table2-5y. Appearance frequencies of the keywords by five-year periods. Table2-10y. Appearance frequencies of the keywords by ten-year periods. (ZIP 8463 kb) [file 12906_2019_2562_MOESM1_ESM.zip › cluster imagesR3.docx]

**Supplementary material**


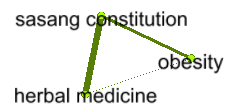


Figure S1. Cluster 1 of research topics.


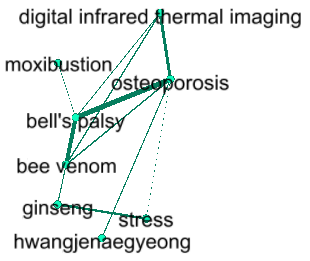


Figure S2. Cluster 2 of research topics.


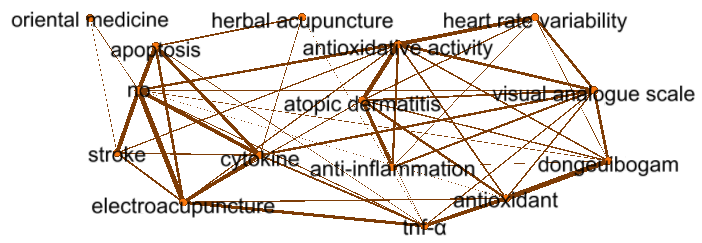


Figure S3. Cluster 3 of research topics.


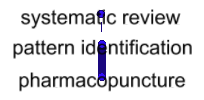


Figure S4. Cluster 4 of research topics.


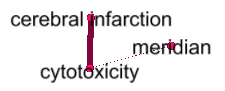


Figure S5. Cluster 5 of research topics.


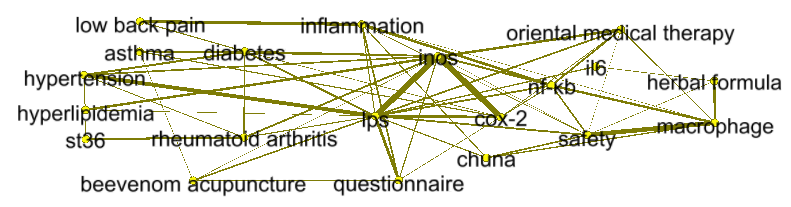


Figure S6. Cluster 6 of research topics.


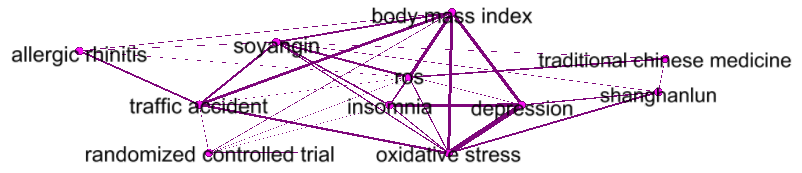


Figure S7. Cluster 9 of research topics.


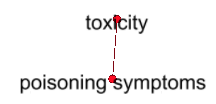


Figure S8. Cluster 10 of research topics.
